# Imatinib therapy for a patient with metastasis of colonic gastrointestinal stromal tumor: report of a case

**DOI:** 10.1007/s12328-013-0365-2

**Published:** 2013-02-18

**Authors:** Takuma Okamura, Tatsuo Kanda, Seiichi Hirota, Atsushi Nishimura, Mikako Kawahara, Keiya Nikkuni

**Affiliations:** 1 Division of Digestive and General Surgery, Niigata University Graduate School of Medical and Dental Sciences, 1-757 Asahimachi-dori, Niigata, 951-8510 Japan; 2 Department of Surgery, Nagaoka Chuo General Hospital, Nagaoka, Japan; 3 Department of Surgical Pathology, Hyogo College of Medicine, Nishinomiya, Japan

**Keywords:** Colon, Gene analysis, GIST, Imatinib, KIT

## Abstract

Gastrointestinal stromal tumors (GISTs) developing in the colon are rare, accounting for <5 % of all GISTs. There are few data on the clinical efficacy of tyrosine kinase inhibitors in colonic GISTs. We report here on an 80-year-old male patient with advanced GIST of the transverse colon. The patient underwent palliative resection of the primary tumor because the disease was associated with multiple liver metastases and peritoneal dissemination. Immunohistochemical analysis of the surgical specimens showed KIT and CD34 expression. Sequence analysis revealed that the tumor harbored deletion mutation at codons 557–558 in exon 11 of the c-*kit* gene. A diagnosis of colonic GIST was made. The patient postoperatively underwent imatinib therapy for the remaining metastatic tumors. Imatinib therapy induced a cyst-like appearance of the liver metastases and stabilized the disease. In the present case, c-*kit* gene analysis was found to be clinically helpful for validating the diagnosis and therapeutic decision making for this rare disease.

## Introduction

Gastrointestinal stromal tumor (GIST) is an uncommon disease, accounting for 0.1–3.0 % of all gastrointestinal neoplasias [1, 2]. GISTs preferentially develop in the stomach and the small bowel, although the tumors can arise anywhere in the digestive tract from the esophagus to the rectum. GISTs originating from the large bowel are rare, accounting for only 5 % of all GISTs [3]. Colonic GIST is even rarer because many of the large bowel GISTs arise in the rectum. Owing to the lack of data on this rare malignancy, the clinical and pathological features of colonic GIST are still unclear.

Imatinib mesylate, a tyrosine kinase inhibitor, shows high clinical efficacy in advanced GIST [4, 5] and is now the standard treatment for unresectable and metastatic GISTs [6]. However, data on the efficacy of imatinib in colonic GIST are scarce only one report written in Japanese is available [7]. The response to imatinib is known to depend on the mutation sites of the c-*kit* gene or the platelet-derived growth factor receptor-alpha gene [8]. However, because data on the mutation rate of the c-*kit* gene and the genotypic profile in colonic GIST are also limited, it remains unknown whether or not colonic GIST is as sensitive as stomach and small bowel GISTs to imatinib.

We experienced a patient with advanced GIST of the transverse colon. The patient underwent palliative resection of the primary tumor and postoperative imatinib therapy. Here, we present the clinical course and our analysis of data on c-*kit* gene mutation in the surgically excised tumor.

## Case report

### Clinical course

**Fig. 1:**
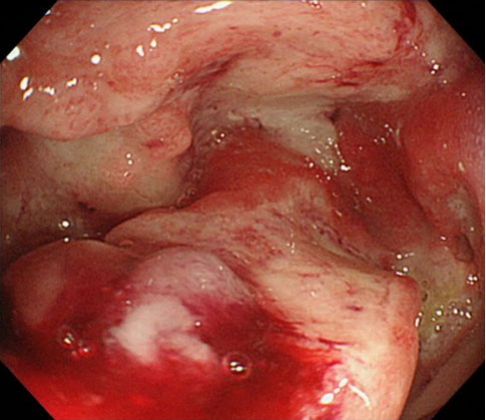
Colonoscopic finding. A large tumor having a central ulceration was found in the transverse colon

An 80-year-old man was diagnosed with a massive tumor having a central ulceration in the transverse colon after undergoing screening colonoscopy (Fig. 1). Computed tomography (CT) revealed that the patient was suffering from multiple metastases in the right hepatic lobe. Biopsy specimens obtained by colonoscopy showed only necrotic debris. Although confirmative diagnosis was not made, our clinical diagnosis was colonic carcinoma associated with multiple liver metastases. After obtaining the patient’s informed consent, we planned surgical resection of both primary colonic tumor and metastatic liver tumors.

Laparotomy revealed many whitish deposits that were widely distributed in the peritoneal cavity. One of the disseminated deposits was sent for pathological examination. A diagnosis of metastasis of sarcomatoid tumors was made on the basis of histological examination using intraoperative frozen section analysis. The patient underwent only partial resection of the transverse colon to confirm the histological diagnosis of the tumor. The small bowel was not involved in the colonic tumor. In addition, no other tumors were found in the gastrointestinal tract. The postoperative course was uneventful. The patient was discharged on postoperative day (POD) 12.

### Pathology and gene analysis

**Fig. 2:**
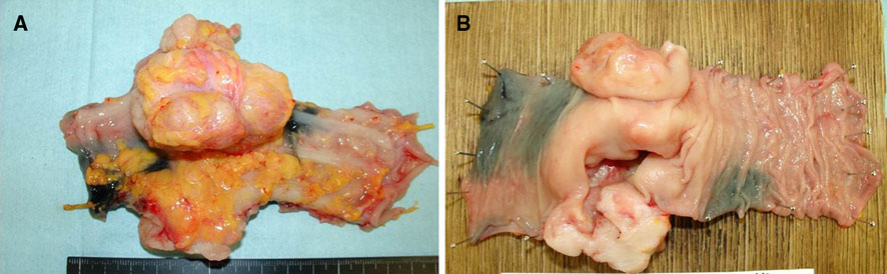
Macroscopic findings of the surgical specimen. **a** The tumor measured 8.0 × 6.0 × 4.0 cm and appeared as an exophytic growth (view from the serosal side). **b** The tumor formed a deep central ulceration (view from the luminal side)

**Fig. 3:**
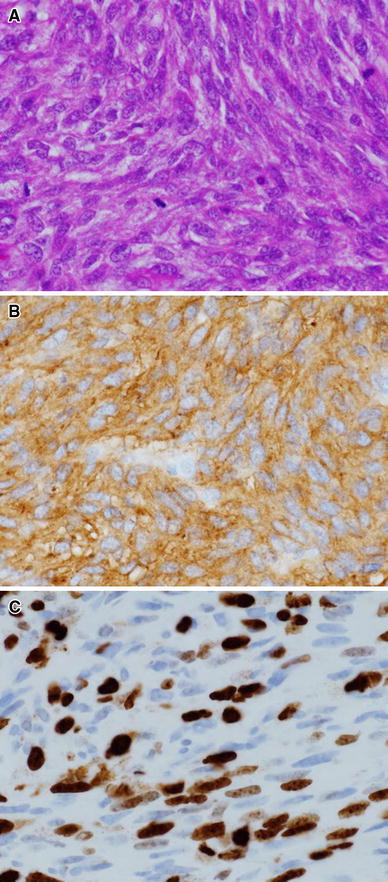
**a** The tumor consisted of solid sheets of spindle tumor cells with moderate atypia (H&E stain, ×400). **b** The tumor cells diffusely showed strong immunoreactivity for KIT (×400). **c** The Ki-67 labeling index was 40 % (×400)

The colonic tumor measured 8.0 × 6.0 × 4.0 cm and appeared as an exophytic growth with a deep central ulceration that opened the colonic lumen (Fig. 2). Microscopically, the tumor consisted of solid sheets of spindle tumor cells with moderate atypia (Fig. 3a). Immunohistochemical analysis revealed that the tumor cells were positive for KIT (Fig. 3b) as well as CD34, and negative for alpha-smooth muscle actin and S-100 protein expression. Based on these histological and immunohistochemical findings, the tumor was diagnosed as a GIST of the transverse colon. The tumor showed markedly high mitotic index, counting 11 in 10 high-power fields. The Ki-67 labeling index was 40 % (Fig. 3c). Lymph node metastasis was also seen in a paracolic node that was surgically picked up.

**Fig. 4:**
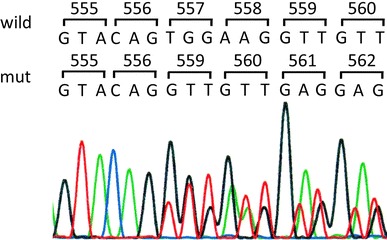
DNA sequence analysis of the *c*-*kit* gene. Sequence analysis revealed heterozygous deletion mutation (TGG AAG) at codons 557–578 in exon 11 of the c-*kit* gene. Nucleotide sequences and corresponding codon numbers are indicated in the *upper* *panel*

To make a confirmative diagnosis of GIST, we conducted mutation analysis of the tumor. DNA was extracted from the formalin-fixed, paraffin-embedded tumor tissues and exons 9, 11, 13, and 17 of the c-*kit* gene were amplified by the polymerase chain reaction. Sequence analysis revealed that the tumor harbored deletion mutation at codons 557–558 in exon 11 of the c-*kit* gene (Fig. 4). A tumor that metastasized to the peritoneum showed the same mutational genotype as the primary tumor.

### Imatinib therapy

**Fig. 5:**
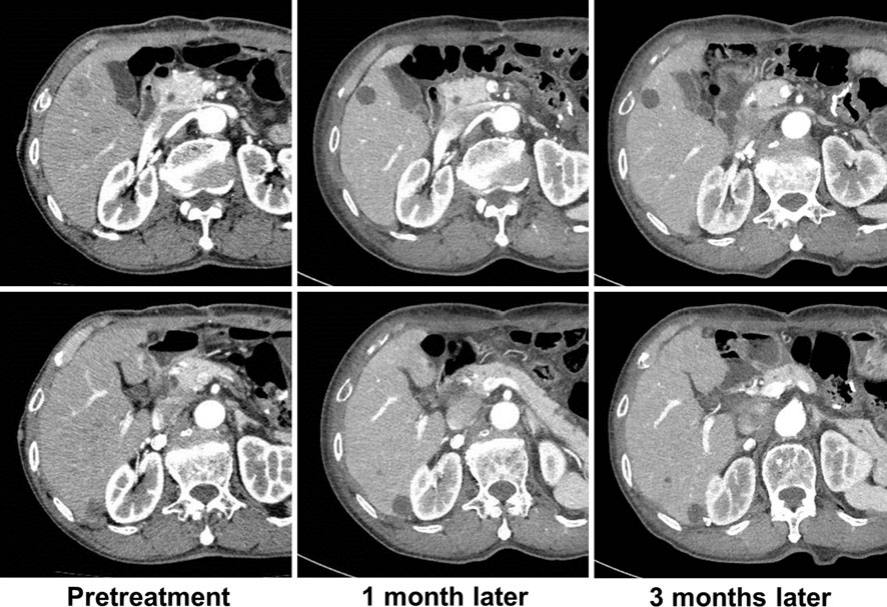
Contrast-enhanced computed tomography (CT) findings. Liver metastases were transformed into sharply circumscribed, non-enhanced homogeneous lesions 1 month after the start of imatinib therapy. CT conducted 3 months after the start of imatinib therapy showed that the response was still maintained. The *upper* *panel* shows a metastasis located in the anterior segment of the liver (S5). The *lower* *panel* shows a metastasis located in the posterior segment of the liver (S6)

To control residual metastatic tumors, the patient was started on imatinib therapy with a daily dose of 400 mg on POD 39. A CT scan conducted 36 days after the start of treatment revealed that all the metastatic tumors of the liver showed a cyst-like appearance, indicating a significant response to imatinib. Peritoneal metastases, which measured no less than 0.5 cm initially, became undetectable. A CT scan performed 86 days after the treatment indicated that the tumors shrank further with a 24 % reduction in size (Fig. 5). The patient is continuing imatinib therapy and living without disease progression so far.

## Discussion

Clinicopathological data on colonic GIST patients are scarce [9]. Only three case series studies concerning colonic GIST are available so far [10–12]. We summarize the results of these three case series studies of colonic GIST in Table 1.

**Table 1:**
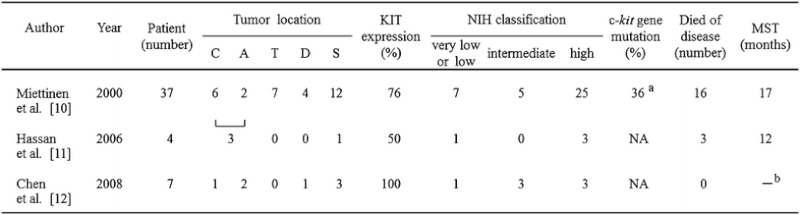
Published case series studies of colonic GIST

*C* cecum, *A* ascending colon, *T* transverse colon, *D* descending colon, *S* sigmoid colon, *MST* median survival time, *NA* not analyzed

^a^Five of 14 tumors were analyzed

^b^All patients were still surviving at the median follow-up time of 27 months

The first one is the study by Miettinen et al. [10]. They analyzed the clinicopathological features of 37 cases of colonic GISTs by reviewing the pathology files of the Armed Forces Institute of Pathology and Helsinki University. The study by Miettinen et al. included a much larger number of patients than the two other studies and thus can be regarded as the basis for consideration of the clinicopathological features of colonic GISTs. In their series, the most common location of colonic GIST was the sigmoid colon, followed by the transverse colon, the cecum, the descending colon, and the ascending colon. The sigmoid colon was also the most common site in a study by Chen et al. [12]. However, in a study by Hassan et al [11], three of four cases were found in the right-sided colon. Shonaka et al. [13], by analyzing case reports of Japanese patients with colonic GISTs, found that six of 14 cases of colonic GIST developed in the sigmoid colon. Based on these case series studies, we can understand that the sigmoid colon is the most common site of colonic GISTs.

Concerning pathological features, the positive rates of KIT expression in colonic GISTs ranged from 50−100 % by immunohistochemical analysis. The tendency in immunohistochemical features was not specified by the previous studies. Meanwhile, as regards the malignant potential of tumors, all the three studies showed high rates of high-risk GIST, ranging from 43−75 %. A high malignant potential may be one of the pathological features of colonic GISTs. As a reflection of the high malignant potential, the prognoses for colonic GIST patients seemed to be poor overall. In Miettinen et al.’s study of the 28 patients who were followed up, 16 (57 %) died of the disease and the median survival time (MST) was 17 months. In the study by Hassan et al., three of four patients died of GIST within 20 months and MST was 12 months. Meanwhile, no patient died of the disease in the study by Chen et al. at the time of reporting, although the median follow-up time was as short as 27 months. More studies with long follow-up periods are needed to conclude that colonic GIST is a GIST subset that is characterized by poor prognosis.

The present case was positive for lymph node involvement. Lymph node metastasis of GIST is rare. Dematteo et al. [14] found lymph node metastasis in six (3 %) of 200 cases of GISTs of the digestive tract, and Miettinen et al. [10] reported no lymph node metastasis in 37 cases of colonic GISTs. In the present case, as the colonic tumor was associated with peritoneal metastasis, lymph node metastasis might be a reflection of the high grade malignancy of this tumor.

Molecularly targeted therapy with imatinib mesylate is the standard treatment for unresectable and metastatic GISTs. Furthermore, imatinib therapy is now recommended as postoperative adjuvant therapy for patients with high-risk GISTs [15], as ample clinical evidence has been accumulated [16, 17]. Thus, it is clinically important to reveal whether or not imatinib is as clinically effective in colonic GIST patients as in patients with stomach and small bowel GISTs. Recently, a case series study addressed the efficacy of imatinib against large bowel GISTs by analyzing exclusively patients with rectal GISTs and not including patients with colonic GISTs [18]. In that study, of the 16 patients who underwent preoperative imatinib therapy, 12 (75 %) showed partial response and the remaining four, stable disease.

We searched published cases of colonic GIST patients who underwent imatinib therapy using PubMed and Japana Centra Revuo Medicina databases. The keywords were ‘colon’, ‘GIST’, and ‘imatinib’. Hits were carefully examined and only a single case report written in Japanese was found to fit the prerequisites [7]. In that case report, a 46-year-old male patient with KIT-positive GIST of the ascending colon underwent postoperative imatinib therapy because the circumferential tumor margins were histologically positive. Despite ongoing imatinib therapy, disseminated peritoneal metastases appeared 9 months after the surgery and the patient died of the disease 1 month later. In that case, the patient had no target lesion and disease relapse occurred as early as 9 months after the surgery, making it difficult to evaluate the effect of imatinib. In our case, the hepatic metastases showed a significant response although it was evaluated as stable disease on the basis of RECIST criteria. Thus, the present case report is the first to indicate that imatinib was effective for colonic GIST.

Gene analysis is important not only for the precise diagnosis of GISTs but also for deciding the best treatment strategy for GIST patients, because the effect of imatinib is closely related to the mutation type of the c-*kit* gene in the tumor. An exploratory study of patients enrolled in a phase II study (B2222) [8] revealed that the response rates were 83.5 % in exon 11 mutations, 47.8 % in exon 9 mutations, and 0 % in the wild type. These findings should underscore the importance of adjuvant therapy in colonic GIST patients because there are no reliable data on the clinical efficacy of imatinib in these patients. Unfortunately, genotype data are also scarce in colonic GIST. Miettinen et al.’s study [10] was the only study that conducted c-*kit* gene analysis. They reported c-*kit* mutations in five (36 %) of 14 colonic GISTs. The genotypes were a point mutation at codon 560 in two cases and a point mutation at codon 559, an in-frame deletion at codons 556–557, and an in-frame deletion at codons 557–558 in one case each. In the present case, an in-frame deletion mutation at codons 557–558 in exon 11 was found. These exon-11 mutations in colonic GISTs are similar to those in stomach and small bowel GISTs [19], suggesting that fundamentally similar molecular alterations occur in colonic GIST.

In summary, we presented a case of a patient with a transverse colon GIST. Mutation analysis showed an in-frame deletion in exon 11 of the c-*kit* gene and confirmed the diagnosis of colonic GIST. The patient underwent imatinib therapy after palliative resection, and the treatment yielded a significant response. This is the first report demonstrating that imatinib was effective for the metastasis of colonic GIST. There are a limited number of colonic GIST patients in a single institution. To acquire fundamental clinical knowledge of colonic GIST, the accumulation of case reports is essential.

## References

[1] Fletcher CD, Berman JJ, Corless C, Gorstein F, Lasota J, Longley BJ (2002). Diagnosis of gastrointestinal stromal tumors: a consensus approach. Hum Pathol.

[2] Crosby JA, Catton CN, Davis A, Couture J, O’Sullivan B, Kandel R (2001). Malignant gastrointestinal stromal tumors of the small intestine: a review of 50 cases from a prospective database. Ann Surg Oncol.

[3] Miettinen M, Lasota J (2001). Gastrointestinal stromal tumors—definition, clinical, histological, immunohistochemical, and molecular genetic features and differential diagnosis. Virchows Arch.

[4] Demetri GD, von Mehren M, Blanke CD, Van den Abbeele AD, Eisenberg B, Roberts PJ (2002). Efficacy and safety of imatinib mesylate in advanced gastrointestinal stromal tumors. N Engl J Med.

[5] Nishida T, Shirao K, Sawaki A, Koseki M, Okamura T, Ohtsu A (2008). Efficacy and safety profile of imatinib mesylate (ST1571) in Japanese patients with advanced gastrointestinal stromal tumors: a phase II study (STI571B1202). Int J Clin Oncol.

[6] Nishida T, Hirota S, Yanagisawa A, Sugino Y, Minami M, Yamamura Y (2008). Clinical practice guidelines for gastrointestinal stromal tumor (GIST) in Japan: English version. Int J Clin Oncol.

[7] Yamaguchi N, Shiomi M, Tojima Y, Kamiya S, Watanabe K, Otsuji H (2008). A case of gastrointestinal stromal tumor of ascending colon with a lymph node metastasis (in Japanese with English abstract). Nihon Shokakigeka Gakkaizasshi.

[8] Heinrich MC, Corless CL, Demetri GD, Blanke CD, von Mehren M, Joensuu H (2003). Kinase mutations and imatinib response in patients with metastatic gastrointestinal stromal tumor. J Clin Oncol.

[9] Miettinen M, Lasota J (2006). Gastrointestinal stromal tumors: pathology and prognosis at different sites. Semin Diagn Pathol.

[10] Miettinen M, Sarlomo-Rikala M, Sobin LH, Lasota J (2000). Gastrointestinal stromal tumors and leiomyosarcomas in the colon: a clinicopathologic, immunohistochemical, and molecular genetic study of 44 cases. Am J Surg Pathol.

[11] Hassan I, You YN, Dozois EJ, Shayyan R, Smyrk TC, Okuno SH (2006). Clinical, pathologic, and immunohistochemical characteristics of gastrointestinal stromal tumors of the colon and rectum: implications for surgical management and adjuvant therapies. Dis Colon Rectum.

[12] Chen CW, Wu CC, Hsiao CW, Fang FC, Lee TY, Che FC (2008). Surgical management and clinical outcome of gastrointestinal stromal tumor of the colon and rectum. Z Gastroenterol.

[13] Shonaka T, Misawa K, Kikuchi K, Takeda K, Okawa Y, Ogawa Y, et al. A case report of gastrointestinal mesenchymal tumor which is detected by perforation peritonitis. (in Japanese with English abstract). Nihon Shokakigeka Gakkaizasshi. 2007;40:1938–43. Jpn J Gastroenterol Surg.

[14] DeMatteo RP, Lewis JJ, Leung D, Mudan SS, Woodruff JM, Brennan MF (2000). Two hundred gastrointestinal stromal tumors: recurrence patterns and prognostic factors for survival. Ann Surg.

[15] Demetri GD, von Mehren M, Antonescu CR, DeMatteo RP, Ganjoo KN, Maki RG (2010). NCCN Task Force report: update on the management of patients with gastrointestinal stromal tumors. J Natl Compr Canc Netw.

[16] Dematteo RP, Ballman KV, Antonescu CR, Maki RG, Pisters PW, Demetri GD (2009). Adjuvant imatinib mesylate after resection of localised, primary gastrointestinal stromal tumour: a randomised, double-blind, placebo-controlled trial. Lancet.

[17] Joensuu H, Eriksson M, Hall KS, Hartmann JT, Pink D, Schütte J (2012). One vs three years of adjuvant imatinib for operable gastrointestinal stromal tumor: a randomized trial. JAMA.

[18] Jakob J, Mussi C, Ronellenfitsch U, Wardelmann E, Negri T, Gronchi A, et al. Gastrointestinal stromal tumor of the rectum: results of surgical and multimodality therapy in the era of imatinib. Ann Surg Oncol. 2012 (Epub ahead of print). 10.1245/s10434-012-2647-1 22965573

[19] Miettinen M, Lasota J (2006). Gastrointestinal stromal tumors: review on morphology, molecular pathology, prognosis, and differential diagnosis. Arch Pathol Lab Med.

